# Evolution of Chloroplast J Proteins

**DOI:** 10.1371/journal.pone.0070384

**Published:** 2013-07-23

**Authors:** Chi-Chou Chiu, Lih-Jen Chen, Pai-Hsiang Su, Hsou-min Li

**Affiliations:** 1 Institute of Molecular Biology, Academia Sinica, Taipei, Taiwan; 2 Institute of Tropical Plant Sciences, National Cheng Kung University, Tainan, Taiwan; National Taiwan University, Taiwan

## Abstract

Hsp70 chaperones are involved in multiple biological processes and are recruited to specific processes by designated J domain-containing cochaperones, or J proteins. To understand the evolution and functions of chloroplast Hsp70s and J proteins, we identified the Arabidopsis chloroplast J protein constituency using a combination of genomic and proteomic database searches and individual protein import assays. We show that Arabidopsis chloroplasts have at least 19 J proteins, the highest number of confirmed J proteins for any organelle. These 19 J proteins are classified into 11 clades, for which cyanobacteria and glaucophytes only have homologs for one clade, green algae have an additional three clades, and all the other 7 clades are specific to land plants. Each clade also possesses a clade-specific novel motif that is likely used to interact with different client proteins. Gene expression analyses indicate that most land plant-specific J proteins show highly variable expression in different tissues and are down regulated by low temperatures. These results show that duplication of chloroplast Hsp70 in land plants is accompanied by more than doubling of the number of its J protein cochaperones through adding new J proteins with novel motifs, not through duplications within existing families. These new J proteins likely recruit chloroplast Hsp70 to perform tissue specific functions related to biosynthesis rather than to stress resistance.

## Introduction

The heat shock protein 70 kD (Hsp70) family chaperones have been shown to function in many biological processes including protein folding, protein translocation, protein complex assembly and degradation of misfolded proteins. Hsp70s located inside chloroplasts also perform many functions. In green algae analyzed, chloroplast Hsp70 is encoded by a single gene and has been shown to be involved in photoprotection and repair of photodamaged photosystem II [1]. In all the land plants analyzed, chloroplasts contain more than one Hsp70 [2]–[4]. Different functions have been suggested for land plant chloroplast Hsp70s, including folding and assembly of individual proteins or protein complexes [5]–[8]. One of the chloroplast Hsp70s in *Physcomitrella patens* is important for protein translocation into chloroplasts [3]. Arabidopsis has two chloroplast Hsp70s, cpHsc70-IV and cpHsc70-V (originally named cpHsc70-1 and cpHsc70-2, respectively; cp stands for chloroplast and the Roman numerals stand for the chromosome where the gene is located. We changed the names in order to avoid confusion with different mutant alleles of each locus). Both are important for protein import into chloroplasts [4] and cpHsc70-IV is also important for conferring thermotolerance to germinating seeds [9]. It is not known whether the functions of algal chloroplast Hsp70 are still preserved in land plant chloroplasts or whether the identified functions of land plant chloroplast Hsp70s are unique to land plants.

One way to investigate the functions of Hsp70 and to reveal new involvement of Hsp70 is to characterize its J domain-containing cochaperones, otherwise known as the J proteins. Hsp70 is recruited to specific functions by association with designated J proteins [10]. One can follow the evolution of J proteins to identify the addition or loss of Hsp70 participation in certain processes. J domain is a four-helix structure of approximately 70 amino acids with an invariant histidine-proline-aspartic acid (HPD) tripeptide motif located in the loop between helix II and III. The HPD motif is necessary for stimulating ATP hydrolysis by Hsp70. Some J proteins also deliver substrate proteins to Hsp70. J proteins are ubiquitous in prokaryotes and eukaryotes. The prototype of J proteins is *E. coli* DnaJ, which contains four structural domains: an N-terminal J domain, followed by a Gly/Phe-rich domain, a Zn^2+^-finger domain and a less conserved C-terminal domain. Consequently, J proteins are classified into three types. Type-I J proteins contain all four domains described for *E. coli* DnaJ. Type-II J proteins contain the J domain and the Gly/Phe-rich domain. Type-III J proteins have only the J domain in common with *E. coli* DnaJ [11]. Some J proteins contain additional structural domains not present in *E. coli* DnaJ, such as transmembrane domains, tetratricopeptide repeat (TPR) domains and ferredoxin (Fd) domains [12], [13]. Some proteins contain a J domain-like structure but lack the conserved HPD tripeptide motif. These proteins are referred to as J-like proteins.

In *Chlamydomonas reinhardtii,* five chloroplast J proteins, named CDJ1 to CDJ5, have been analyzed. They are suggested to function in processes ranging from biogenesis of thylakoid membranes, translation, to mRNA stability [12], [14]–[16]. Several J proteins have also been identified in pea and Arabidopsis chloroplasts, including one type-I J protein (PCJ1, [17]) and four type-III J proteins [18]–[21]. Arabidopsis J8, J11 and J20 (called DJC22, DJC23 and DJC26, respectively, in [22] and the current work) have been suggested to be involved in optimization of photosynthesis [18]. CRRJ (called NdhT in [23] and DJC75 in [22] and the current work) is a thylakoid membrane protein and is essential for the activity of the chloroplast NAD(P)H dehydrogenase (NDH) complex functioning in cyclic electron transport [21]. It is not known which higher plant chloroplast J proteins have homologs in green algae and cyanobacteria or which J proteins are newly evolved and thus their functions may be unique to land plants. It is also not known how multiplication of chloroplast *HSP70* genes in land plants has affected the number of chloroplast J proteins. For example, J proteins may have also duplicated within families, or new families of J protein may have evolved to direct chloroplast Hsp70 to new functions.

As one of the first steps toward characterizing the evolution and functions of chloroplast Hsp70s and J proteins, we investigated the constituency, domain structure and evolutionary origins of Arabidopsis chloroplast J proteins. Because predictions of chloroplast-targeting transit peptides tend to have a higher false positive rate and proteomic analyses tend to only identify proteins of higher abundance, we combined all available transit peptide prediction and proteomic databases and then verified all candidate proteins by individual chloroplast protein import assays. Although laborious, this approach will provide the best-possible complete list of chloroplast J proteins. Only with a near complete list of members can analyses on the evolution of chloroplast J proteins be performed. We found that Arabidopsis chloroplasts contain at least 19 J proteins. Phylogenetic analyses showed that these 19 J proteins could be classified into 11 clades. All 11 clades are conserved in land plants, and more than half of them are present only in land plants, suggesting many new functions have evolved for land plant chloroplast Hsp70s. Analyses of expression patterns indicate that land plant-specific J proteins in general show highly variable expression levels in different tissues and are down regulated by low temperatures.

## Results

### Nomenclature of Arabidopsis J Proteins

Four systems have been used for naming Arabidopsis J proteins in the literature. The first and most commonly used is the prefix “AtJ” followed by an Arabic numeral that stands for the order of appearance of the proteins in publications. For example, the first and second Arabidopsis J proteins published were named AtJ1 and AtJ2 [24], [25]. Later, a report searching the then newly finished Arabidopsis genome identified 89 J proteins in Arabidopsis [26]. This report used a different nomenclature: “atDj(A, B, C)x”, in which the “at” stands for *Arabidopsis thaliana*, “Dj” stands for DnaJ, A, B, and C represent type I, II and III J proteins, respectively, and “x” is a number from 1 to 89 which mirrors the original system of ordering by the appearance of the J proteins in published works, regardless of the type of protein. However, most subsequent papers continue to use the “AtJx” system for Arabidopsis J proteins. In 2009, Rajan & D’Silva searched the Arabidopsis genome again and found that there were 116 J proteins in Arabidopsis [27]. Although these investigators also used the “atDj(A, B, C)x” acronym, in their work the “x” stood for a serial number within each type of J proteins. For example, atDjA3 and atDjB9 in Miernyk (2001) were renamed atDjA1 and atDjB1, respectively, by Rajan & D’Silva (2009). Recently, Finka *et al.* (2011) revised the total number of J proteins from 116 to 105, and renamed J proteins as “DJ(A, B, C)x” with the numeric number “x” re-sorted [22]. This correction was based on the finding that 8 of the J proteins listed by Rajan & D’Silva contain transposable elements and another 3 were given two independent numbers. In this article, we have decided to follow Finka *et al*. (2011); however, for clarity Table 1 lists all the J proteins tested in this study and their corresponding names according to all the nomenclature systems.

**Table 1:** Putative chloroplast J proteins of Arabidopsis analyzed in this work.

J-protein^a^	Locus name	Protein accession	Molecular mass^b^ (residues)	Predicted subcellular localization^c^	Chloroplast proteomics^d^	Import into chloroplasts^e^	Former name^f^	Former name^g^	alternative name^h^	Identified homolog in other species^i^
DJA4	At3g17830	NP_188410	57.3 (517)	m,p/m/−/m	+/−/+	+	atDjA4	atDjA54 (AtJ54)		
DJA5	At4g39960	NP_568076	48 (447)	p/p/−/p	+/+/+	+	atDjA5	atDjA24 (AtJ24)		
DJA6	At2g22360	NP_565533	47.8 (442)	c,p/p/−/p	+/−/+	+	atDjA6	atDjA26 (AtJ26)		CDJ1
DJA7	At1g80030	BAH19589	53.8 (500)	p,s/p/−/p	+/−/+	+	atDjA7	atDjA52 (AtJ52)		PCJ1, CDJ6
DJC22	At1g80920	NP_178207	18.3 (163)	p/p/−/p	−/−/−	+	atDjC25	atDjC8 (AtJ8)		
DJC23	At4g36040	NP_195328	17.9 (161)	p/p/−/p	−/−/−	+	atDjC26	atDjC11 (AtJ11)		
DJC24	At2g17880	NP_179378	17.7 (160)	p,s/p/−/p	−/−/−	+	atDjC27	atDjC41 (AtJ41)		
DJC26	At4g13830	AAF24498	23.4 (197)	p/p/−/p	−/−/−	+	atDjC29	atDjC20 (AtJ20)		
DJC31	At5g12430	NP_568276	129.2 (1165)	−/p/p,n/p	−/−/−	+	atDjC34	–	TPR16	
DJC42	At5g27240	NP_198076	124.5 (1104)	n/n/−/n	−/−/+	–	atDjC45	atDjB47 (AtJ47)		
DJC62	At2g41520	NP_850351	122.8 (1108)	n/n/p,n/n	−/−/−	+	atDjC72	atDjB67 (AtJ67)	TPR15	
DJC65	At1g77930	NP_565163	31.9 (271)	m,p/m/−/m	−/−/−	+	atDjC75	atDjC55 (AtJ55)		
DJC66	At3g13310	NP_187939	17.4 (157)	p/m/−/m	−/−/−	+	atDjC76	atDjC38 (AtJ38)		
DJC69	At5g18140	NP_197315	37.6 (333)	p,n/m/−/m	−/−/−	+	atDjC79	atDjC56 (AtJ56)		
DJC72	At2g18465	NP_849977	30.7 (268)	−/p/−/c	−/−/−	+	atDjC82	–		
DJC73	At5g59610	NP_200769	30.3 (268)	c,p/m/−/m	−/−/−	+	atDjC83	atDjB42 (AtJ42)		CDJ2
DJC75	At4g09350	NP_192673	28.5 (249)	m/p/−/p	+/−/−	+	atDjC85	atDjC25 (AtJ25)	CRRJ, NdhT	
DJC76	At5g23240	NP_197715	51.8 (465)	m,p,n/m/−/m	−/−/−	+	atDjC86	atDjC17 (AtJ17)		CDJ5
DJC77	At2g42750	NP_565982	38.8 (344)	m,p,n/p/−/p	−/+/−	+	atDjC87	atDjC18 (AtJ18)		CDJ3, CDJ4
DJC78	At4g07990	NP_567329	27.8 (230; 347^j^)	−/−/−/p^j^	−/−/−	–	atDjC88	–		
DJC82^k^	At3g05345	NP_001154591	27.6 (244)	p^k^	−/−/−	+	–	–		

a Nomenclature according to Finka et al. (2011).

b Molecular mass in kD.

c Predicted subcellular localization listed in Miernyk (2001)/Rajan and D’Silva (2009)/Prasad et al. (2010)/Finka et al. (2011); progame used by Miernyk (2001): Psort, TargetP, Predotar and Mitoprot; by Rajan and D’Silva (2009): Mitoprot, ChloroP, SUBA, TargetP and Wolf psort; by Prasad et al. (2010): SUBA, iPSORT, MitoPred, Mitoprot II, MultiLoc, PeroxP, Predotar, SubLoc, TargetP and Wolf psort; by Finka et al. (2011): the Uniport database; c, cytosol; m, mitochondria; p, plastid; n, nucleus; s, secretory pathway; –, the gene is not yet annotated as a J protein in the publication.

d PPDB database (http://ppdb.tc.cornell.edu/)/plport database (http://www.plprot.ethz.ch/)/AT_CHLORO database (http://www.grenoble.prabi.fr/at_chloro/).

e Results from this work (Figure 1).

f Nomenclature according to Rajan and D’Silva (2009).

g Nomenclature according to Miernyk (2001); commonly used AtJx naming system is also shown in parentheses.

h TPR15 and TPR16 from Pradad et al. (2010); CRRJ from Yamamoto et al. (2011); NdhT from Ifuku et al. (2011).

i PCJ1 from Schlicher and Soll (1997); CDJ1 from Willmund et al. (2008); CDJ2 from Liu et al. (2005); CDJ3 to CDJ5 from Dorn et al. (2010); CJD6 from GenBank (Accession number: EDO96593).

j According to the incorrect old annotation (see text).

k Newly named J protein in this study, suggested to be localized in plastids by TAIR.

### Nineteen J Proteins were Imported into Chloroplasts

Using various prediction algorithms, Miernyk (2001) and Rajan & D’Silva (2009) suggested that 18 J proteins might have chloroplast-targeting transit peptides. In addition, we searched various published chloroplast proteomes and found that DJC42, although predicted as a nuclear protein [27], was detected in chloroplasts [28]. DJC62, a TPR domain-containing J protein, was also predicted to localize in chloroplasts by Pradas *et al*. (2010). In addition, At3g05345, named DJC82 in our current study, is a newly annotated J protein that is predicted to be localized in chloroplasts (TAIR, http://www.arabidopsis.org/). To determine how many of these 21 putative chloroplast J proteins are localized to chloroplasts, protein import assays were performed. The 21 J proteins were synthesized and labeled with [^35^S]Met by in vitro translation, and then incubated with isolated pea chloroplasts under import conditions. After import, a portion of the chloroplasts was further treated with thermolysin to remove surface-associated precursor proteins. Our initial results showed that lower-molecular-weight mature proteins were produced after the import of 15 of the J proteins analyzed (Figure 1A) and their mature proteins were also thermolysin-resistant after import. For all 15 proteins, no protein with the same molecular weight as the mature protein was detected from thermolysin-treated precursor proteins without import, indicating that the thermolysin-resistant mature protein was produced as a result of import into chloroplasts, not as a result of the intrinsic protease resistance of the proteins.

**Figure 1:**
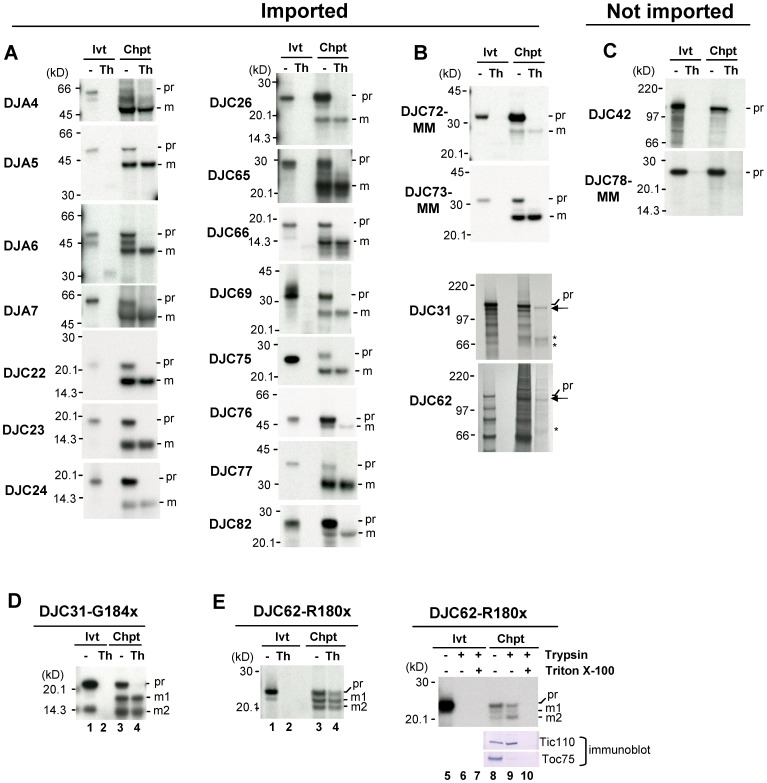
Nineteen J proteins were imported into chloroplasts. (A) Fifteen J proteins that were imported into chloroplasts. (B) Chloroplast import of DJC72MM, DJC73MM, DJC31 and DJC62. (C) DJC42 and DJC78MM were not imported into chloroplasts. (D) Chloroplast import of DJC31-G184x. (E) Chloroplast import of DJC62-R180x. In vitro-translated [^35^S]Met-labeled precursor proteins were incubated with isolated pea chloroplasts under import conditions. Part of the precursor proteins without import (Ivt) or re-isolated intact chloroplasts after import (Chpt) were further treated with thermolysin (Th) or trypsin. Sample for lanes 7 and 10 of (E) were trypsin treated in the presence of 0.1% of Triton X-100. Samples were analyzed by SDS-PAGE followed by fluorography. For each precursor panel, the Ivt lanes (with and without protease treatment) contain the same amount of precursor, and the Chpt lanes contain the same amount of proteins. The Ivt lanes contained 0.9% of the in vitro-translated proteins added to the import reactions shown in the Chpt lanes, except for DJC62, in which the Ivt lanes represent 0.4% of in vitro-translated proteins added to the import reactions. pr, precursor form; m, mature form. m1 and m2, different-sized mature proteins produced after the import of DJC31-G184x and DJC62-R180x. Samples from lane 8 to 10 in (E) were also analyzed by immunoblotting for Toc75 and Tic110 as controls.

Four of the J proteins analyzed (DJC42, DJC72, DJC73 and DJC78) produced no thermolysin-resistant proteins after import. Among them, DJC72 only has the initial methionine, and DJC73 and DJC78 only contain methionines within the N-terminal half of their polypeptides. If these three J proteins were imported into chloroplasts, their mature protein might not be seen after the removal of the N-terminal transit peptide. Constructs with two methionines added to the C terminus of these three J proteins were therefore generated (DJC72MM, DJC73MM and DJC78MM). Lower-molecular-weight mature protein could indeed be detected after the import of DJC72MM and DJC73MM (Figure 1B), but not DJC78MM (Figure 1C). DJC78 was previously annotated as a protein of 347 residues [27] and contained a predicted N-terminal transit peptide. Recently, based on EST information, a new annotation from TAIR shows that DJC78 has only 230 residues, lacking the N-terminal 117 residues of the previous annotation. We also found that the coding sequence of the newly annotated DJC78 could be amplified by RT-PCR, but the 347-residue DJC78 from the previous annotation could not (data not shown). These data suggest that the annotation of previous 347-residue DJC78 with a predicted transit peptide was incorrect. No attempt was made to retest DJC42 (Figure 1C) by methionine addition because it contains 1,104 residues and the last methionine is at residue 1,033. DJC42 was identified in a chloroplast proteome experiment by only one peptide [28]. Therefore association of DJC42 with chloroplasts might be a result of contamination.

DJC31 is a large TPR domain-containing J protein of approximately 129 kD. Its import efficiency into chloroplasts was low and three proteins were seen after its import (Figure 1B, DJC31, arrow and asterisks). Of these, the protein with the highest molecular weight (Figure 1B, DJC31, arrow) was extremely close in size to the precursor protein. If this protein is the mature protein, DJC31 must have a short transit peptide. If one of the two lower-molecular-weight proteins (Figure 1B, DJC31, asterisks) is the mature protein, DJC31 must have a transit peptide larger than 50 kD. To confirm that DJC31 could be imported into chloroplasts and to clarify the size of its transit peptide, a C-terminally truncated clone of DJC31, DJC31-G184x, was created by mutating the glycine of residue 184 to a stop codon. After the import of DJC31-G184x, which is about 22 kD, two mature proteins with sizes between 14 to 18 kD were produced (Figure 1D, lanes 3 and 4, m1 and m2). This result confirmed that DJC31 has a short transit peptide of about 5 kD. The relationship between the two mature proteins m1 and m2 is not known but both were only produced after import and were localized within chloroplasts as shown by their resistant to thermolysin treatment (Figure 1D, lane 4). We also do not know the origins of the two smaller proteins produced after the import of the full-length DJC31 (Figure 1B, DJC31, asterisks), but they were most likely degraded forms of mature DJC31. Interestingly, similar results were seen after the import of the other TPR domain-containing J protein DJC62, which has even poorer efficiency of in vitro import. Two proteins were seen after the import of DJC62, with one very close in size to the precursor and the other about 50 kD smaller (Figure 1B, DJC62, arrow and asterisk). We thus generated a C-terminally truncated clone, DJC62-R180x, by mutating the arginine of residue 180 to a stop codon, and performed import experiments. After thermolysin treatment of the chloroplasts after import, one protein the same size as the precursor, and two lower-molecular-weight mature proteins were produced (Figure 1E, lanes 3 and 4, pr, m1 and m2). To further confirm the location of these three proteins, chloroplasts after import were treated with trypsin (Figure 1E, lanes 5 to 10). Trypsin is more effective in removing outer membrane proteins but still will not penetrate the inner membrane. The result showed that all three imported proteins were resistant to trypsin (Figure 1E, lane 9) and suggested that they were located inside the inner membrane. They were degraded if the trypsin treatments were performed in the presence of Triton X-100, indicating that their resistance was due to membrane protection, not protein aggregation (Figure 1E, lane 10). The effectiveness of the trypsin treatment was shown by the degradation of the outer membrane protein Toc75 and the resistance of the inner membrane protein Tic110. The presence of the precursor form as one of the imported products indicated that the transit peptide of DJC62 is not always removed after import or the efficiency of processing was low in the in vitro import experiments.

In summary, our data show that Arabidopsis has at least 19 J proteins localized in chloroplasts (Figure 1 and Table 1). All 19 have predicted transit peptides. In addition, consistent with the stroma location of the two chloroplast Hsp70s [9], [29], none of the J proteins has the bipartite transit-peptide structure typical of thylakoid luminal proteins.

### The Nineteen Chloroplast J Proteins in Arabidopsis Can Be Classified into 11 Clades

**Figure 2:**
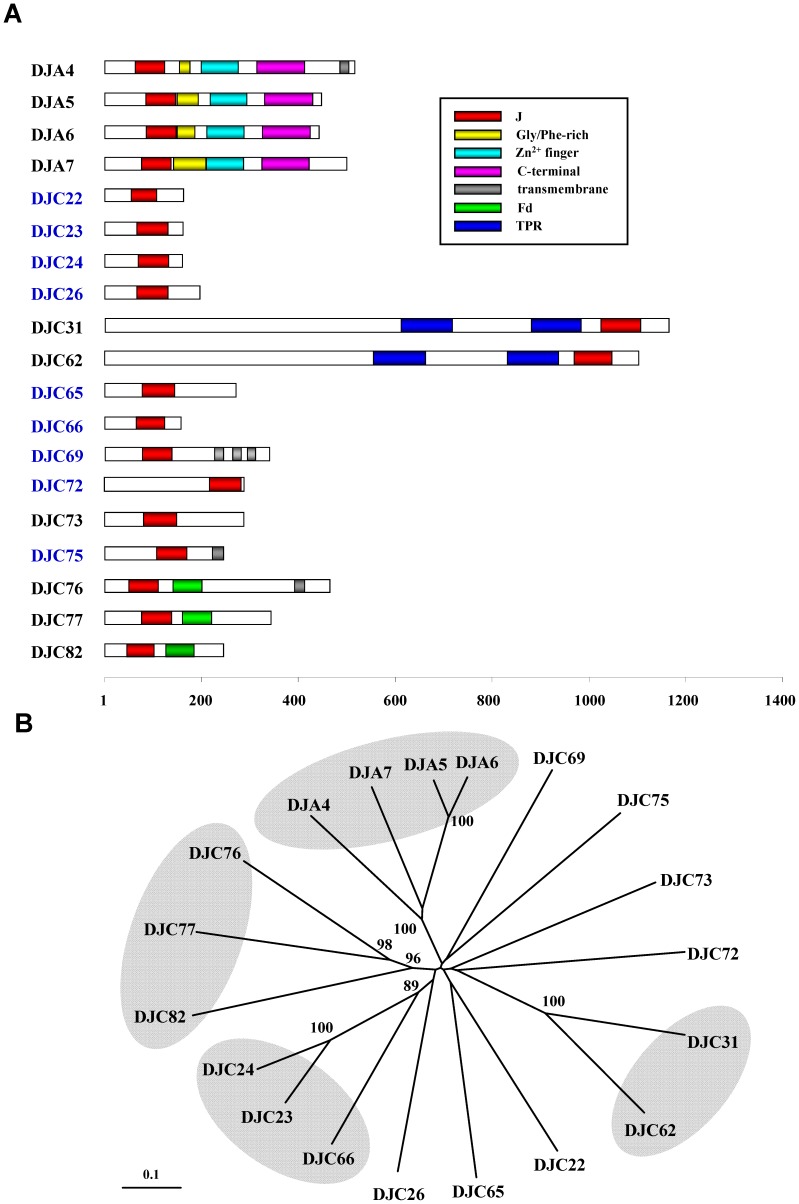
Domain structure and phylogenetic tree of the 19 Arabidopsis chloroplast J proteins. (A) Schematic representations of domain structures of the 19 Arabidopsis chloroplast J proteins. Each bar is drawn to scale according to the number of amino acids, shown at the bottom. Names of land plant-specific J proteins are written in blue. J, J domain; Gly/Phe-rich, glycine/phenylalanine-rich domain; Zn^2+^ finger, zinc-finger domain; C-terminal, C-terminal domain; Fd, ferredoxin domain; TRP, tetratricopeptide repeat domain. (B) Phylogenetic relationship of the 19 Arabidopsis chloroplast J proteins. A neighbor-joining tree was constructed using multiple sequence alignments from peptide sequences of the 19 chloroplast J proteins. Bootstrap analyses were computed with 1,000 replicates, and the values of percentage larger than 85 are shown on the branches. Grouping of four clades (shown with gray background) had at least 85% bootstrap support.

Among the 19 chloroplast J proteins, four are type-I J proteins. The other 15 are all type-III J proteins. Some of the chloroplast J proteins contain additional known structural domains (Figure 2A). Both DJC31 and DJC62 have two TPR domains. DJC76, DJC77 and DJC82 have an Fd domain. DJA4, DJC69, DJC75 and DJC76 have one or more predicted transmembrane domains. Phylogenetic analysis revealed that the 19 J proteins could be classified into 11 clades, consisting of 4 groups and 7 singletons (Figure 2B). All of the type-I J proteins, DJA4, DJA5, DJA6 and DJA7, fall into one group. The other three groups include the two TPR domain-containing J proteins (DJC31 and DJC62), the three smallest chloroplast J proteins (DJC23, DJC24 and DJC66) and the three Fd domain-containing J proteins (DJC76, DJC77 and DJC82). Other than the type-I J proteins, no homologues are found for the rest of the 10 clades in Arabidopsis.

### 10 of the 11 Clades of Chloroplast J Proteins are Conserved in Flowering Plants

To investigate whether the 11 clades of chloroplast J proteins identified in Arabidopsis were conserved in other flowering plants, we performed BLASTP searches against genome databases of rice (*Oryza sativa*), soybean (*Glycine max*) and wine grape (*Vitis vinifera*) using polypeptide sequences of the 19 Arabidopsis chloroplast J proteins as queries. As shown in Table 2, ortholog(s) for all of the 11 clades could be identified except that DJC69 ortholog could not be found in rice. We further searched for DJC69 homolog in other available monocot databases but still did not find DJC69 homologs in any monocots, suggesting that DJC69 has been lost in monocots.

**Table 2:** Homologs of Arabidopsis chloroplast J protein in other plant species.

*Synechocystis sp. PCC 6803*	*Cyanophora paradoxa*	*Chlamydomonas reinhardtii*	*Physcomitrella patens*	*Selaginella moellendorffii*	*Vitis vinifera*	*Glycine max*	*Arabidopsis thaliana*	*Oryza saiva*
Sll0897	Contig38447	CDJ1	Pp1s81_162V6.1	g167519	GSVIVT01006041001	Glyma05g31080	DJA4	LOC_Os02g56040
		CDJ6	Pp1s93_165V6.1		GSVIVT01008471001	Glyma07g11690	DJA5	LOC_Os03g12236
			Pp1s136_80V6.1		GSVIVT01017355001	Glyma08g14290	DJA6	LOC_Os05g26902
			Pp1s225_24V6.1			Glyma11g38040	DJA7	LOC_Os05g26914
			Pp1s386_29V6.1			Glyma13g44310		LOC_Os05g26926
						Glyma15g00950		
						Glyma18g01960		
		CDJ2	Pp1s82_38V6.1	g8874^e^	GSVIVT01024914001	Glyma13g41360	DJC73	LOC_Os03g60790
			Pp1s137_288V6.1			Glyma15g04040		
		CDJ3	Pp1s54_91V6.1	g413357	GSVIVT01001992001	Glyma03g39790	DJC76	LOC_Os01g53020
		CDJ4	Pp1s198_9V6.2	g73652	GSVIVT01001994001	Glyma06g42800	DJC77	LOC_Os04g57880
		CDJ5	Pp1s297_5V6.1		GSVIVT01026228001	Glyma09g42020	DJC82	LOC_Os05g45350
			Pp1s18_322V6.1		GSVIVT01020748001	Glyma12g15560		LOC_Os05g33010b
			Pp1s40_24V6.1		GSVIVT01000053001	Glyma12g33970		
			Pp1s54_85V6.1			Glyma13g36560		
						Glyma19g13280		
						Glyma20g00450		
		Cre02.g108800	Pp1s29_56V6.1	g171422	GSVIVT01005044001	Glyma11g10100	DJC31	(LOC_Os05g31056/LOC_Os05g31062)^a^
					GSVIVT01031545001	Glyma12g02420	DJC62	
			Pp1s17_154V6.1	**g8923** c	**GSVIVT01015991001**	**Glyma07g02480**	**DJC22**	**LOC_Os06g44160**
			**Pp1s17_157V6.1**			Glyma08g23540		
			Pp1s35_131V6.1					
			Pp1s100_202V6.1					
			Pp1s100_218V6.1					
			Pp1s110_50V6.1					
			Pp1s112_204V6.1					
			Pp1s159_108V6.1					
			Pp1s227_127V6.1					
			Pp1s354_16V6.1					
			**Pp1s204_80V6.1**	**g100874** d	GSVIVT01009924001	**Glyma01g37090**	**DJC23**	**LOC_Os08g43490**
					**GSVIVT01014782001**	Glyma02g05400	DJC24	
					GSVIVT01024057001	Glyma08g44900	DJC66	
						Glyma11g08190		
						Glyma14g01440		
						Glyma16g23750		
						Glyma18g08040		
			**Pp1s91_206V6.1**	**g441708**	**GSVIVT01006408001**	**Glyma08g11580**	**DJC26**	**LOC_Os01g01160**
			Pp1s160_132V6.1		GSVIVT01021014001			
			**Pp1s60_191V6.1**	**g73318**	**GSVIVT01008959001**	**Glyma06g11260**	**DJC65**	**LOC_Os07g43330**
			**Pp1s106_74V6.1**	**g448880**	**GSVIVT01012773001**	**Glyma08g26020**	**DJC75**	**LOC_Os11g10990**
						Glyma12g00300		
			**Pp1s77_267V6.1**	**g412304**	**GSVIVT01033405001**	**Glyma19g28880**	**DJC69**	
			Pp1s57_179V6.1			Glyma16g04540		
					**GSVIVT01026590001**	Glyma04g39420	**DJC72**	LOC_Os04g59060
						Glyma06g15480		LOC_Os05g01590
						**Glyma09g19770**		

a rice DJC31 homolog, OsDJC31 (accession: BK008486), covering previously annotated LOC_Os05g31056 and LOC_Os05g31062, was re-annotated according to the alignment with DJC31 homologs from Arabidopsis and other monocots (Figure S2).

b rice DJC82 homolog, OsDJC82 (accession: BK008487), was re-annotated according to the alignment with DJC82 homologs from other plant species (Figure S3).

c *Selaginella* DJC22 homolog, SmDJC22 (accession: BK008488), was re-annotated according to the alignment with DJC22 homologs from s other plant species (Figure 4).

d *Selaginella* DJC23 homolog, SmDJC23 (accession: BK008489), was re-annotated according to the alignment with DJC23 homologs from other plant species (Figure 3).

e *Selaginella* DJC73 homolog, SmDJC73 (accession: BK008490), was re-annotated according to the alignment with DJC73 homologs from other plant species (Figure S4).

Note: Homolog shown in boldface is the representative from each species for the 7 clades of land plant-specific chloroplast J protein aligned in Figures 3 and 4.

Classification of chloroplast J proteins into 11 clades is still evident when the phylogenetic analysis is expanded from Arabidopsis to the other flowering plants analyzed (Figure S1). The Fd domain-containing J-protein clade (DJC76 clade) can be further divided into three subclades, DJC76, DJC77 and DJC82, when more homologs were included. These results suggest that the 11 clades of chloroplast J proteins identified from Arabidopsis are conserved in flowering plants with the exception that DJC69 homolog does not exist in monocots.

### Evolutionary Origins of the Chloroplast J Proteins

We next investigated where the chloroplast J proteins appeared in the evolutionary lineage. Polypeptide sequences of the 19 Arabidopsis chloroplast J proteins were used to perform TBLASTN searches against the genomes of cyanobacterium *Synechocystis* sp. PCC 6803, glaucophyte *Cyanophora paradoxa*, 7 green algae (three *Ostreococcus,* two *Micromonas pusilla, Chlamydomonas reinhardtii* and *Volvox carteri*), and two lower land plant species, the moss *Physcomitrella patens* and lycopod *Selaginella moellendorffii*. As shown in Table 2 and Table S1, only type-I J-protein homologs exist in cyanobacterium *Synechocystis* sp. PCC 6803 and glaucophyte *Cyanophora paradoxa.* Homologs for an additional three clades, the TPR domain-containing DJC31 clade, the Fd domain-containing DJC76 clade, and the DJC73 clade, are present in all the green algae analyzed (Table S1). Six more clades were found in *Physcomitrella* and *Selaginella*, finally the DJC72 clade appears only in flowering plants and the DJC69 clade was then lost in monocots.

Homologs of all six *Chlamydomonas* chloroplast J proteins previously reported are present in all the land plants analyzed (Table 2), suggesting that functions identified for *Chlamydomonas* chloroplast Hsp70 and J proteins are most likely preserved in higher plant chloroplasts. These *Chlamydomonas* chloroplast J proteins comprise three of the four clades we identified: the type-I J proteins (*Chlamydomonas* CDJ1 and CDJ6), the Fd domain-containing DJC76 clade (CDJ3 to 5) and the DJC73 clade (CDJ2). Our searches identified additionally locus *Cre02.g108800* of *Chlamydomonas* as encoding a homolog of the TPR domain-containing DJC31 clade. The protein encoded by *Cre02.g108800* contains a large, less conserved, N-terminal region, followed by two TPR domains and a C-terminal J domain, resembling Arabidopsis DJC31 and DJC62. All of the green algae analyzed contain the same four clades (Table S1). These results suggest that green algae only possess part of the higher plant chloroplast J protein constituency. However, we cannot exclude the possibility that green algae contain some yet unidentified chloroplast J proteins and these proteins have been lost in higher plants.

### Only the Type-I J Protein from *Synechocystis* is Retained

*Synechocystis* sp. PCC 6803 genome has 7 genes encoding J proteins (Table S2) [30]. Our search results indicate that only the type-I J protein in Arabidopsis chloroplasts were derived from the cyanobacterium. To confirm that all other *Synechocystis* J proteins were not retained, we used the polypeptide sequences of these 7 J proteins to perform BLASTP searches against the Arabidopsis genome. Only the type-I J protein of *Synechocystis* sp. PCC 6803, Sll0897, has homologs in Arabidopsis (Table 1). Although *sll1384* encodes a protein that is predicted to contain one TRP domain, the protein has no sequence similarity to Arabidopsis DJC31 or DJC62 and its J domain was located N terminal to the TPR domain, rather than C terminal as in Arabidopsis DJC31 and DJC62. Therefore it is unlikely that Arabidopsis DJC31 and DJC62 have evolved from Sll1384. To further confirm that the 6 *Synechocystis* J proteins have been lost, the 6 *Synechocystis* J proteins were searched against the glaucophyte *Cyanophora paradoxa* genome. Glaucophytes have chloroplasts with cyanobacterial appearance [31], [32], and have been shown to be the earliest divergence in Plantae [33]. Again our results showed that only the type-I J protein homologs were found in the *Cyanophora paradoxa* genome and no homologs were found for the other 6 *Synechocystis* J proteins. Thus, our result suggests that during the endosymbiotic process, only type-I J protein was retained, and the other six cyanobacterial J proteins were no longer retained, at least since glaucophytes.

### The Seven Land Plant-Specific J-Protein Clades May Serve Different Functions

All the 7 clades of land plant-specific chloroplast J proteins are relatively small with no additional known domains other than the J domain (Figure 2A, names labeled in blue). We were interested in knowing whether they still contain some clade-specific motifs that suggest they play different functions. Their sequences from six land plant species were aligned. If a species has multiple family members for a particular clade, a representative gene was selected. As shown in Figures 3 and 4, all 7 clades have at least one clade-specific highly conserved motif in addition to the J domain. All, except DJC72, have the clade-specific motifs located close to the C terminus (Figure 3). In agreement with the fact that it is the newest addition since flowering plants, sequences of DJC72 from different species are similar across the entire polypeptide even in the transit peptide regions. The high degree of sequence identity across different species in the clade-specific motif suggests that the motif has conserved function from moss to flowering plants, and is most likely used to interact with a specific client protein or a protein that recruits the J protein to a specific location within chloroplasts. These highly conserved motifs are clade-specific, suggesting that each clade interacts with a different protein. In the DJC22 clade, rice does not seem to share the conserved C-terminal motif found in *Physcomitrella*, *Selaginella* and the dicot plants (Figure 4). However, when we retrieved DJC22 homologs from other monocots, we found that the C-terminal domain of all monocot DJC22s is highly conserved (Figure 4). It is possible that the client protein for DJC22 is conserved from *Physcomitrella* and *Selaginella* to dicots. In monocots, the structure of the client protein, and thus the client-recognition motif in DJC22, may have further evolved to adapt to some monocot-specific physiology.

**Figure 3:**
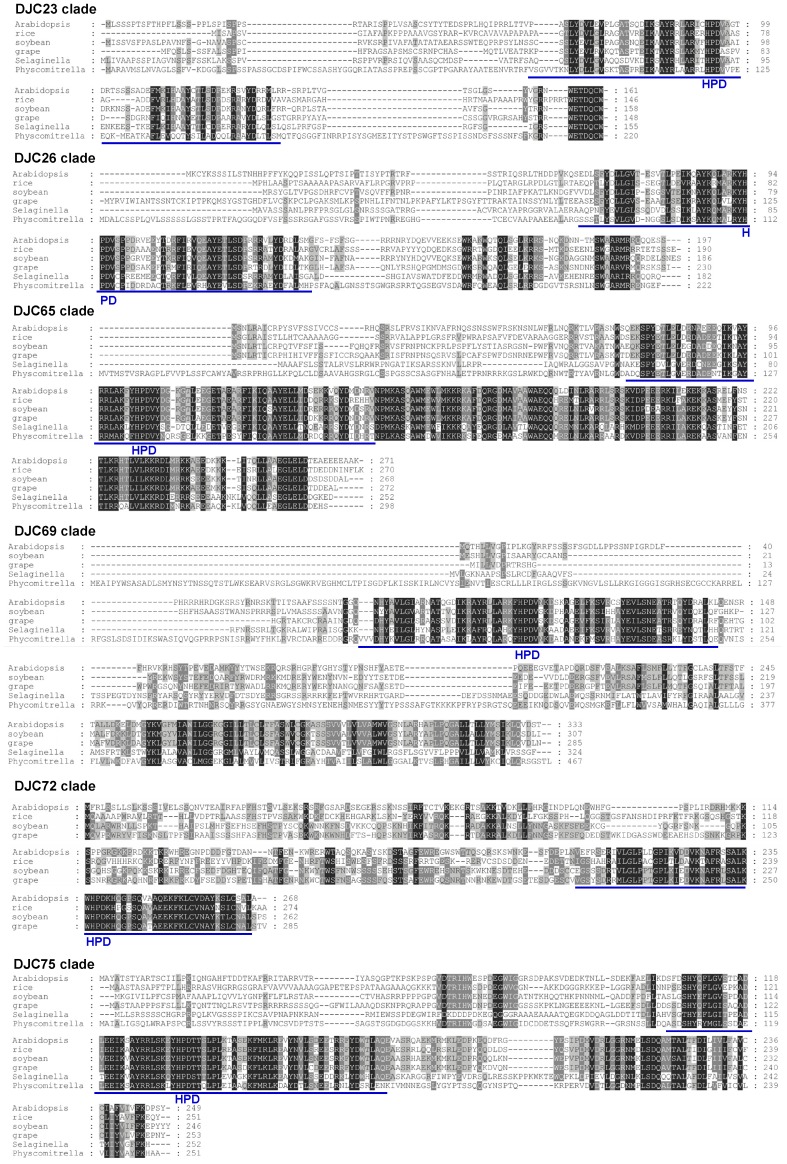
Land plant specific chloroplast J proteins contain highly conserved clade-specific motifs. Sequence alignments of 6 of the land plant-specific chloroplast J-protein clades. Representative genes (bold typed in Table 2) from each species were aligned. The J domain is underlined with a blue line. The position of the HPD tripeptide is indicated.

**Figure 4:**
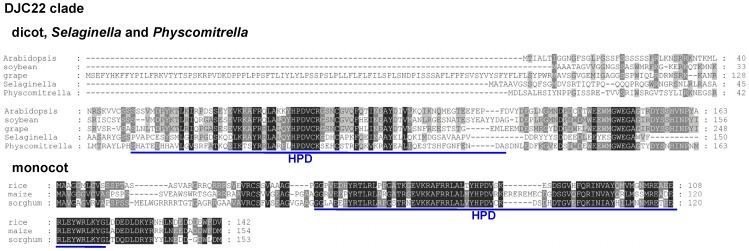
DJC22 proteins from monocots have distinct clade-specific motif from dicots and lower land plants. Sequence alignments of DJC22 homologs from dicot plants, *Selaginella* and *Physcomitrella*, and sequence alignments of DJC22 homologs from monocot plants. Representative genes (bold typed in Table 2) from each species, and homologs from maize (GRMZM2G086841) and sorghum (Sb04g025270) were aligned. The J domain is underlined with a blue line. The position of the HPD tripeptide is indicated.

### Gene Expression Patterns of Chloroplast J Proteins in Arabidopsis

**Figure 5:**
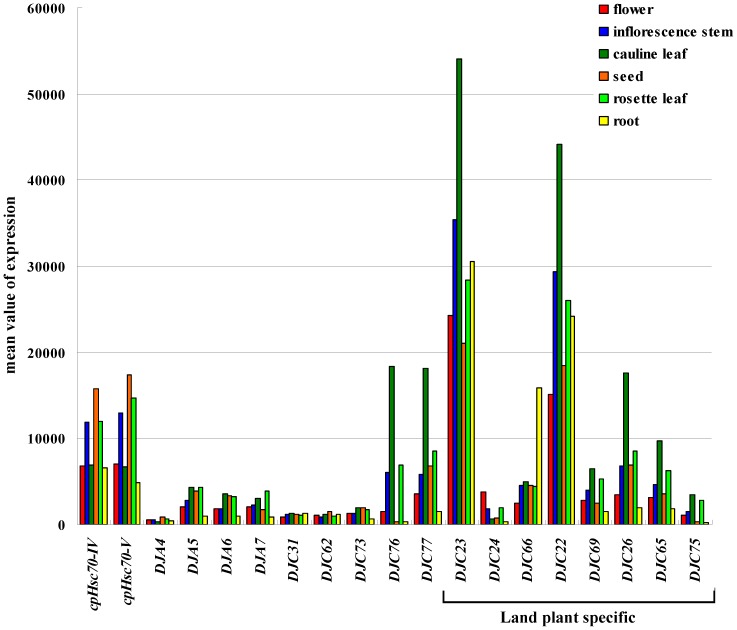
Tissue-specific expression patterns of chloroplast J-protein genes in Arabidopsis. Expression of 17 chloroplast J-protein genes and two *cpHsc70s* genes in various tissues was retrieved using Genevestigator. Mean values of expression level after normalization were plotted. Land plant-specific J proteins are indicated.

As a first step toward investigating the functions of the chloroplast J proteins, gene expression levels of the Arabidopsis chloroplast J proteins in various tissues was retrieved from the public Affymetrix microarray database using Genevestigator (www.genevestigator.com) [34]. DJC72 and DJC82 do not yet have probe sets in the Affymetrix gene chips and thus were not included in the analyses. As shown in Figure 5, in general, land plant-specific chloroplast J proteins show larger variations in expression levels in different tissues, suggesting that they have some tissue-specific functions or that their amount needs to be adjusted according to plastid types. The two Fd domain-containing J proteins, DJC76 and DJC77, although not land plant-specific, also show variable expression in different tissues with the highest expression in leaves. It is likely that they have preserved their function from algal chloroplasts and thus mostly function in chloroplasts of leaves. DJC75 (also named CRRJ or NdhT) is land plant-specific and is expressed almost exclusively in green tissues, suggesting that it has a function unique to land-plant chloroplasts. Indeed DJC75 is essential for the activity of the NDH complex functioning in cyclic electron transport [21]. NDH complex is not found in green algae [23]. DJC23 has the highest expression level among all Arabidopsis chloroplast J proteins. Interestingly, the other two members of the same clade, DJC24 and DJC66, are expressed predominantly in flowers and roots respectively, two highly specialized organs of higher plants. These expression patterns suggest that this clade may have some functions that require tissue-specific adjustment of different isoforms. Other J proteins with homologs present in green algae, including the four type-I J proteins, the two TPR domain-containing J proteins (DJC31 and DJC62) and DJC73, all show more uniform and lower expression levels in the major tissues we selected, suggesting that they serve some primordial constitutive functions in all plastid types.

### Regulation of *cpHsc70* and J-Protein Gene Expression by Abiotic Stresses

**Figure 6:**
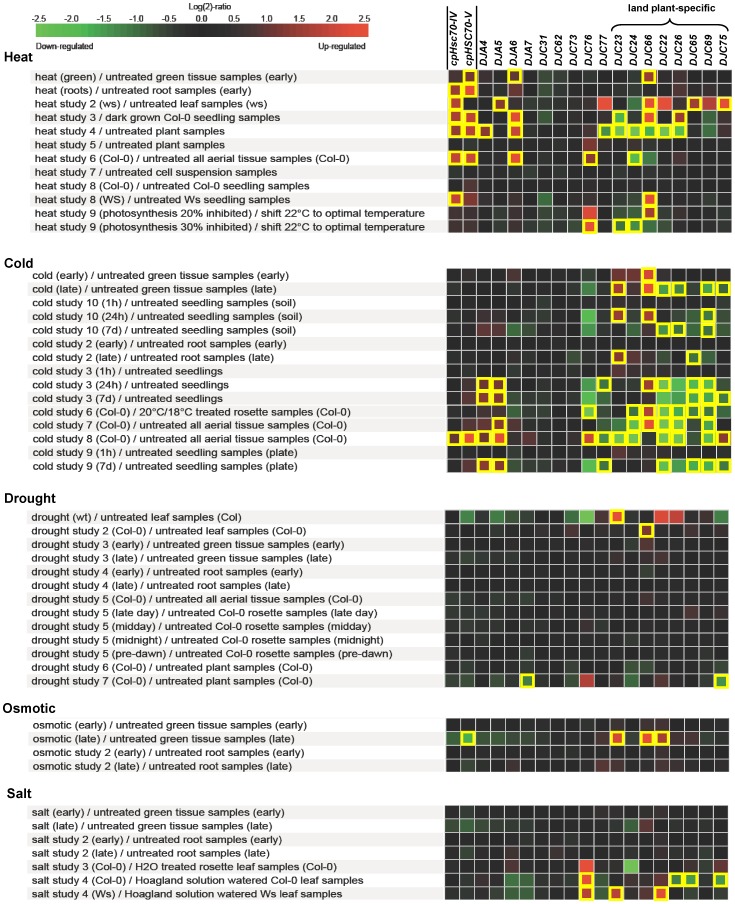
Regulation of chloroplast J-protein and the two *cpHsc70* genes by various abiotic stresses. Expression ratios (log2 values of stress-treated/controls, as indicated by red and green colored squares) of 17 chloroplast J-protein genes and two *cpHsc70* genes under five different stresses in wild-type Arabidopsis were analyzed using Genevestigator. Filters for fold change and *p*-value were set to>|2| and <0.05, respectively, for consistent stress regulation (marked with open yellow boxes).

As sessile organisms, plants have to adapt to the environmental changes. To determine if some of the chloroplast J proteins are involved in the adaptation, expression patterns of chloroplast J-protein and the two *cpHsc70* genes under abiotic stresses, such as heat, cold, drought, osmotic and salt stresses, were analyzed using Genevestigator. As show in Figure 6, when the filters for fold change and *p*-value were set to |2| and <0.05, respectively, heat becomes the only stress that can up-regulate the expression of the *cpHsc70* genes in multiple experiments. *DJA6* and *DJC66* were also up-regulated by heat stress in multiple experiments, suggesting that DJA6 and DJC66 may function together with cpHsc70s in thermotolerance. *DJA4*, *DJA5*, *DJC23* and *DJC66* were up-regulated by cold stress. They may recruit cpHsc70 to assist the folding of some cold-labile proteins [35]. *DJC77*, and many land plant-specific J-protein genes, such as *DJC24*, *DJC22*, *DJC26*, *DJC65*, *DJC69* and *DJC75*, were down-regulated by cold stress. *DJC76* is the only gene regulated by salt stress. Drought and osmotic stresses do not seem to have a significant effect on the expression of chloroplast J-protein genes since no gene was affected significantly in multiple experiments.

## Discussion

We show here that at least 19 Arabidopsis J proteins are localized in chloroplasts. Since our starting pool was J proteins with a predicted chloroplast-targeting transit peptide and J proteins identified in various plastid proteomes, J proteins with non-canonical chloroplast targeting signals and present in very low amounts would be missed in this analysis. Thus the exact number of chloroplast J proteins can be expected to be even higher. However 19 J proteins already make chloroplasts the organelle with the highest number of confirmed J proteins.

Our analyses indicate that three new clades of J proteins were added since green algae. New J proteins may have evolved to recruit cpHsc70 to new functions, or to processes already present in lower organisms but not yet involving Hsp70. For example, CDJ2 of *Chlamydomonas* has been suggested to assist the assembly and disassembly of the vesicle-inducing protein 1 (VIPP1) oligomers [15], which is also present in cyanobacteria [36]. Since there is no CDJ2 homolog in cyanobacteria (Table 2), VIPP1 may work with proteins other than Hsp70 and J proteins in cyanobacteria.

Our comparison of the numbers of J proteins in *Chlamydomonas* and Arabidopsis chloroplasts (Table 3) suggest that when the number of chloroplast Hsp70-encoding genes increased from one to at least two in the transition from green algae to land plants, the number of genes encoding chloroplast J proteins also have more than doubled by adding seven new clades with novel clade-specific motifs. It will be very interesting to identify the new client proteins interacting with these new J proteins. Surprisingly, we had expected the land plant-specific J proteins to function in stress-related conditions like drought, which would be expected to affect land plants more severely than algae. However, gene expression analyses suggest that only DJC66 may be involved in heat and cold stress tolerance. Most land plant-specific J proteins are down regulated by cold treatments. No chloroplast J proteins could be suggested to be involved in drought tolerance, suggesting that drought tolerance may not involve cpHsc70. Down-regulations of many land plant-specific J proteins by cold stress were observed in the late response (after cold treatment ≥24 hours), not in the early stage (1-hour cold treatment) (Figure 6). It has been shown in cold-responsive transcriptome analyses, the majority of genes down-regulated by cold stress in the late response are genes related to metabolism [37]. Hence it is possible that chloroplast J proteins down regulated by cold treatments may function in folding and assembly of proteins and protein complexes involved in metabolism.

**Table 3:** Number of Hsp70 and J-protein homologs in various organisms, and in chloroplasts of Arabidopsis and *Chlamydomonas*.

		Hsp70	J protein	reference
Organism	*Escherichia coli*	3	6	[39]
	*Synechocystis* sp.PCC 6803	3	7	[30]
	*Saccharomyces* *cerevisiae*	14	22	[10]
	*Homo sapiens*	17	41	[10], [38]
	*Chlamydomonas reinhardtii*	7	63	[16], [40]
	*Arabidopsis* *thaliana*	14	106	[41] and this work
Chloroplast	*Chlamydomonas* chloroplasts	1	7	[40] and this work
	*Arabidopsis*chloroplasts	2	19	[41] and this work

In prokaryotes, yeast and animals, the ratio between the number of J protein-encoding genes and the number of Hsp70-encoding genes is about 2, and this ratio has increased to around 9 since *Chlamydomonas* and has remained similar in all higher plants (Table 3, [10], [16], [30], [38]–[41]). The incorporation of a cyanobacterium as a new organelle may have incurred many new levels of coordination, for example, regulation of the expression of nuclear genes encoding chloroplast proteins, photorespiration, and other crosstalks between chloroplasts and other organelles. These additional needs may have been accommodated by fusing the J domain to a motif that can bind new client proteins and then tapping into the abundant Hsp70 system for protein homeostasis maintenance and protection.

All the J proteins, except the type-I J protein, from cyanobacteria have been lost during the endosymbiotic process. This suggests that type-I J proteins probably play some primordial role, like assisting in thermotolerance. Functions played by cyanobacterial type-II and type-III J proteins may no longer be required by chloroplasts as intracellular organelles. Type-II J proteins may also have been lost because the functions of type-I and type-II J proteins are partially redundant [30], [42]. The identification of 19 Arabidopsis chloroplast J proteins and the analyses of their evolutionary origins will provide useful leads for finding J proteins assisting chloroplast Hsp70 in specific functions.

## Materials and Methods

### Plant Materials, Chloroplast Isolation and Protein Import into Chloroplasts

Pea (*Pisum sativum* cv. Little Marvel) seedlings were grown as described [19], and 6- to 9-day-old seedlings were harvested for chloroplast isolation. Chloroplast isolation, protein import into isolated chloroplasts and thermolysin treatment of chloroplasts after import were performed as described [43]. Import reactions were performed at room temperature for 25 min with 3 mM ATP. Trypsin treatment of chloroplasts after import was performed as described [44].

### Plasmid Constructions and in vitro Translation of Precursor Proteins

For the 21 cDNA clones encoding J proteins analyzed in this study, 10 clones were requested from the Arabidopsis Biological Resource Center (DJA5, DJC23, DJC24, DJC26, DJC73, DJC76, DJC77, and DJC78; http://abrc.osu.edu/) [45], the French Plant Genomic Resource Center (DJC75; http://cnrgv.toulouse.inra.fr/) or RIKEN BioResource Center (DJC62; http://www.brc.riken.jp/) [46]–[48] (Table S3). The cDNA fragments of the other 11 clones were amplified by PCR from Arabidopsis leaf first-strand cDNA. The cDNA fragments for DJA4, DJA5 and DJA7 were subcloned into the plasmid pCR-Blunt II-TOPO (Invitrogen) and the rest were subcloned into pSP72 (Promega). Construction of DJC22 (AtJ8) was described previously [19]. DJC73MM, DJC31-G184x and DJC62-R180x were amplified by PCR using DJC73, DJC31and DJC62 as the template, respectively. DJC72MM and DJC78MM were generated by QuikChange site-directed mutagenesis kit (Stratagene) using DJC72 and DJC78 as the template, respectively. The names and sequences of the primers used are listed in Tables S3 and S4. All precursor proteins were in vitro translated by the TNT coupled wheat germ extract system (Promega) using various RNA polymerases (Table S3).

### Sequence Analysis and Construction of Phylogenetic Tree

To search for homologs of Arabidopsis chloroplast J proteins in rice (*Oryza sativa*; GenBank v. 171), soybean (*Glycine max*; GenBank v. 181) and grape (*Vitis vinifera*; GenBank v. 179), reciprocal BLASTP searches were performed. In the first query, protein sequences of the 19 chloroplast J proteins were used as queries to search the databases in PlantGDB (http://www.plantgdb.org/) and Phytozome (v. 7.0; http://www.phytozome.net/), which collect and reorganize the most updated sequence information for most of the sequenced plant species. The *E*-value for BLAST was set to 1e-10. Sequences of putative homologs were retrieved and used as the second query to perform BLASTP searches against the Arabidopsis database. If the most similar homolog in Arabidopsis was exactly the Arabidopsis J-protein sequence used as the first query, the sequence of the second query was selected as a homolog. BLASTP results obtained from PlantGDB and Phytozome are the same. Locus names of homologs obtained from PlantGDB are shown in Table 2. Homologs for DJC31 and DJC82 were originally not found in rice, but were found in sorghum and maize and their exon-intron junctions are conserved with the Arabidopsis homologs. We therefore compared the genomic sequence of rice to other higher plants and re-annotated the sequences of the rice homologs for these two J proteins. The peptide sequences of the re-annotated rice DJC31 homolog (original annotation: LOC_Os05g31056/LOC_Os05g31062) and rice DJC82 homolog (original annotation: LOC_Os05g33010) are shown in Figures S2 and S3. The re-annotated rice DJC31 and DJC82 homologs are highly similar to homologs from other plants. To search for chloroplast J-protein homologs in lower plant species, TBLASTN was performed using databases in PlantGDB and Phytozome for *Volvox carteri, Chlamydomonas reinhardtii*, *Physcomitrella patens* and *Selaginella moellendorffii*, Cyanophora genome project (http://cyanophora.rutgers.edu/cyanophora/home.php) for glaucophyte *Cyanophora paradoxa*, and NCBI/BLAST (http://blast.ncbi.nlm.nih.gov/Blast.cgi) for the others. Homologs were identified using the method described above. In *Selaginella*, protein sequences annotated for 3 J-protein homologs located at loci g8923, g100874 and g8874 were short. Through comparison of their genomic sequences with other species, their sequences were re-annotated. The peptide sequences of re-annotated *Selaginella* DJC22 (original annotation: g8923) and DJC23 (original annotation: g100874) homologs are shown in Figures 3 and 4. The peptide sequence of re-annotated *Selaginella* DJC73 homolog (original annotation: g8874) is shown in Figure S4. Multiple sequence alignments were performed using the BLOSUM protein weight matrix and visualized by GeneDoc (v. 2.5; http://www.nrbsc.org/gfx/genedoc/). Phylogenetic trees were constructed using the neighbor-joining method employed by the ClustalX program [49] and visualized by NJplot (v. 2.3; http://pbil.univ-lyon1.fr/software/njplot.html) or TreeView (v. 1.6.6; http://taxonomy.zoology.gla.ac.uk/rod/treeview.html). Domain structures are predicted using InterPro (http://www.ebi.ac.uk/interpro/).

### Expression Pattern Analyses

Tissue-specific expression pattern and stress-regulated gene expression ratios were retrieved from the public Affymetrix microarray database using Genevestigator (www.genevestigator.com).

Nucleotide sequence data of OsDJC31, OsDJC82, SmDJC22, SmDJC23 and SmDJC73 reported in this work are available in the Third Party Annotation Section of the DDBJ/EMBL/GenBank databases under the accession numbers TPA: BK008486–BK008490.

## Supporting Information

Figure S1 **Phylogenetic relationship of chloroplast J proteins from Arabidopsis, rice, soybean and grape.** A neighbor-joining tree was constructed using multiple sequence alignments of full-length polypeptide sequences of genes shown in Table 2. Bootstrap analysis was computed with 1,000 replicates and the values are shown on the branches. The eleven clades classified from analyses of Arabidopsis chloroplast J proteins are marked with different background colors. Three subclades of the Fd domain-containing J-protein clade are indicated.
(PDF)

Figure S2 **Sequence alignment of DJC31 homologs from Arabidopsis (DJC31),** ***Brachypodium distachyon*** **(Bradi2g27160), millet (** ***Setaria italica*** **, SiPROV000210m), maize (GRMZM2G348697), sorghum (Sb09g018680), and the re-annotated rice DJC31, OsDJC31.** OsDJC31 (GenBank accession: BK008486) was re-annotated from the continuous genomic region covered by loci LOC_Os05g31056 and LOC_Os05g31062. The J domain is underlined in blue. The position of the HPD tripeptide is indicated.
(PDF)

Figure S3 **Sequence alignment of re-annotated rice DJC82 homolog with DJC82 homologs from Arabidopsis (DJC82), soybean (Glyma03g39790), grape (GSVIVT01000053001),** ***Selaginella*** **(g73652), and** ***Physcomitrella*** **(Ppls137_288V6.1).** The rice DJC82 homologue, OsDJC82 (GenBank accession: BK008487), was re-annotated from original annotation for locus LOC_Os05g33010. The J domain is underlined in blue. The position of the HPD tripeptide is indicated.
(PDF)

Figure S4 **Sequence alignment of re-annotated** ***Selaginella*** **DJC73 homolog with DJC73 homologs from Arabidopsis (DJC73), rice (LOC_Os03g60790), soybean (Glyma13g41360), grape (GSVIVT01024914001), and** ***Physcomitrella*** **(Ppls137_288V6.1).** The *Selaginella* DJC73 homolog, SmDJC73 (GenBank accession: BK008488), was re-annotated from original annotation for locus g8874. The J domain is underlined in blue. The position of the HPD tripeptide is indicated.
(PDF)

Table S1 **Homologs of Arabidopsis chloroplast J protein in seven green algal genomes.**
(PDF)

Table S2 **J proteins in** ***Synechocystis*** **sp. PCC 6803.**
(PDF)

Table S3 **Information of J-protein clones used in this study.**
(PDF)

Table S4 **Sequences of primers used in this study.**
(PDF)

## References

[1] Schroda M, Vallon O, Wollman FA, Beck CF (1999) A chloroplast-targeted heat shock protein 70 (HSP70) contributes to the photoprotection and repair of photosystem II during and after photoinhibition. Plant Cell 11: 1165–1178. 10368186 10.1105/tpc.11.6.1165 PMC144243

[2] Marshall JS, DeRocher AE, Keegstra K, Vierling E (1990) Identification of heat shock protein hsp70 homologues in chloroplasts. Proc Natl Acad Sci USA 87: 374–378. 2296591 10.1073/pnas.87.1.374 PMC53266

[3] Shi LX, Theg SM (2010) A stromal heat shock protein 70 system functions in protein import into chloroplasts in the moss *Physcomitrella patens* . Plant Cell 22: 205–220. 20061551 10.1105/tpc.109.071464 PMC2828695

[4] Su PH, Li Hm (2010) Stromal Hsp70 is important for protein translocation into chloroplasts. Plant Cell 22: 1516–1531. 20484004 10.1105/tpc.109.071415 PMC2899880

[5] Bonk M, Tadros M, Vandekerckhove J, Al-Babili S, Beyer P (1996) Purification and characterization of chaperonin 60 and heat-shock protein 70 from chromoplasts of *Narcissus pseudonarcissus* . Plant Physiol 111: 931–939. 8754688 10.1104/pp.111.3.931 PMC157912

[6] Chen GG, Jagendorf AT (1994) Chloroplast molecular chaperone-assisted refolding and reconstitution of an active multisubunit coupling factor CF1 core. Proc Natl Acad Sci USA 91: 11497–11501. 7972091 10.1073/pnas.91.24.11497 PMC45258

[7] Madueno F, Napier JA, Gray JC (1993) Newly imported Rieske iron-sulfur protein associates with both Cpn60 and Hsp70 in the chloroplast stroma. Plant Cell 5: 1865–1876. 12271059 10.1105/tpc.5.12.1865 PMC160411

[8] Tsugeki R, Nishimura M (1993) Interaction of homologues of Hsp70 and Cpn60 with ferredoxin-NADP+ reductase upon its import into chloroplasts. FEBS Lett 320: 198–202. 8096466 10.1016/0014-5793(93)80585-i

[9] Su PH, Li Hm (2008) Arabidopsis stromal 70-kD heat shock proteins are essential for plant development and important for thermotolerance of germinating seeds. Plant Physiol 146: 1231–1241. 18192441 10.1104/pp.107.114496 PMC2259073

[10] Kampinga HH, Craig EA (2010) The HSP70 chaperone machinery: J proteins as drivers of functional specificity. Nat Rev Mol Cell Biol 11: 579–592. 20651708 10.1038/nrm2941 PMC3003299

[11] Cheetham ME, Caplan AJ (1998) Structure, function and evolution of DnaJ: conservation and adaptation of chaperone function. Cell Stress Chaperones 3: 28–36. 9585179 10.1379/1466-1268(1998)003<0028:sfaeod>2.3.co;2 PMC312945

[12] Dorn KV, Willmund F, Schwarz C, Henselmann C, Pohl T, et al (2010) Chloroplast DnaJ-like proteins 3 and 4 (CDJ3/4) from *Chlamydomonas reinhardtii* contain redox-active Fe-S clusters and interact with stromal HSP70B. Biochem J 427: 205–215. 20113313 10.1042/BJ20091412

[13] Prasad BD, Goel S, Krishna P (2010) In silico identification of carboxylate clamp type tetratricopeptide repeat proteins in Arabidopsis and rice as putative co-chaperones of Hsp90/Hsp70. PLoS One 5: e12761. 20856808 10.1371/journal.pone.0012761 PMC2939883

[14] Liu C, Willmund F, Golecki JR, Cacace S, Hess B, et al (2007) The chloroplast HSP70B-CDJ2-CGE1 chaperones catalyse assembly and disassembly of VIPP1 oligomers in *Chlamydomonas* . Plant J 50: 265–277. 17355436 10.1111/j.1365-313X.2007.03047.x

[15] Liu C, Willmund F, Whitelegge JP, Hawat S, Knapp B, et al (2005) J-domain protein CDJ2 and HSP70B are a plastidic chaperone pair that interacts with vesicle-inducing protein in plastids 1. Mol Biol Cell 16: 1165–1177. 15635096 10.1091/mbc.E04-08-0736 PMC551482

[16] Willmund F, Dorn KV, Schulz-Raffelt M, Schroda M (2008) The chloroplast DnaJ homolog CDJ1 of *Chlamydomonas reinhardtii* is part of a multichaperone complex containing HSP70B, CGE1, and HSP90C. Plant Physiol 148: 2070–2082. 18931144 10.1104/pp.108.127944 PMC2593681

[17] Schlicher T, Soll J (1997) Chloroplastic isoforms of DnaJ and GrpE in pea. Plant Mol Biol 33: 181–185. 9037170 10.1023/a:1005784115363

[18] Chen KM, Holmstrom M, Raksajit W, Suorsa M, Piippo M, et al (2010) Small chloroplast-targeted DnaJ proteins are involved in optimization of photosynthetic reactions in *Arabidopsis thaliana* . BMC Plant Biol 10: 43. 20205940 10.1186/1471-2229-10-43 PMC2844072

[19] Chiu CC, Chen LJ, Li Hm (2010) Pea chloroplast DnaJ-J8 and Toc12 are encoded by the same gene and localized in the stroma. Plant Physiol 154: 1172–1182. 20841453 10.1104/pp.110.161224 PMC2971597

[20] Orme W, Walker AR, Gupta R, Gray JC (2001) A novel plastid-targeted J-domain protein in *Arabidopsis thaliana* . Plant Mol Biol 46: 615–626. 11516154 10.1023/a:1010665702621

[21] Yamamoto H, Peng L, Fukao Y, Shikanai T (2011) An Src homology 3 domain-like fold protein forms a ferredoxin binding site for the chloroplast NADH dehydrogenase-like complex in Arabidopsis. Plant Cell 23: 1480–1493. 21505067 10.1105/tpc.110.080291 PMC3101538

[22] Finka A, Mattoo RU, Goloubinoff P (2011) Meta-analysis of heat- and chemically upregulated chaperone genes in plant and human cells. Cell Stress Chaperones 16: 15–31. 20694844 10.1007/s12192-010-0216-8 PMC3024091

[23] Ifuku K, Endo T, Shikanai T, Aro EM (2011) Structure of the Chloroplast NADH Dehydrogenase-Like Complex: Nomenclature for Nuclear-Encoded Subunits. Plant Cell Physiol 52: 1560–1568. 21785130 10.1093/pcp/pcr098

[24] Kroczynska B, Zhou R, Wood C, Miernyk JA (1996) AtJ1, a mitochondrial homologue of the Escherichia coli DnaJ protein. Plant Mol Biol 31: 619–629. 8790294 10.1007/BF00042234

[25] Zhou R, Kroczynska B, Hayman GT, Miernyk JA (1995) AtJ2, an arabidopsis homolog of *Escherichia coli* dnaJ. Plant Physiol 108: 821–822. 7610169 10.1104/pp.108.2.821 PMC157405

[26] Miernyk JA (2001) The J-domain proteins of *Arabidopsis thaliana*: an unexpectedly large and diverse family of chaperones. Cell Stress Chaperones 6: 209–218. 11599562 10.1379/1466-1268(2001)006<0209:tjdpoa>2.0.co;2 PMC434402

[27] Rajan VB, D’Silva P (2009) *Arabidopsis thaliana* J-class heat shock proteins: cellular stress sensors. Funct Integr Genomics 9: 433–446. 19633874 10.1007/s10142-009-0132-0

[28] Ferro M, Salvi D, Brugiere S, Miras S, Kowalski S, et al (2003) Proteomics of the chloroplast envelope membranes from *Arabidopsis thaliana* . Mol Cell Proteomics 2: 325–345. 12766230 10.1074/mcp.M300030-MCP200

[29] Ratnayake RM, Inoue H, Nonami H, Akita M (2008) Alternative processing of Arabidopsis Hsp70 precursors during protein import into chloroplasts. Biosci Biotechnol Biochem 72: 2926–2935. 18997426 10.1271/bbb.80408

[30] Duppre E, Rupprecht E, Schneider D (2011) Specific and promiscuous functions of multiple DnaJ proteins in *Synechocystis* sp. PCC 6803. Microbiology 157: 1269–1278. 21292744 10.1099/mic.0.045542-0

[31] Loffelhardt W, Bohnert HJ, BryanP DA, Weber K (1997) The cyanelles of *Cyanophora paradoxa* . Crit Rev Plant Sci 16: 393–413.

[32] Steiner JM, Loffelhardt W (2002) Protein import into cyanelles. Trends Plant Sci 7: 72–77. 11832278 10.1016/s1360-1385(01)02179-3

[33] Reyes-Prieto A, Bhattacharya D (2007) Phylogeny of nuclear-encoded plastid-targeted proteins supports an early divergence of glaucophytes within Plantae. Mol Biol Evol 24: 2358–2361. 17827169 10.1093/molbev/msm186

[34] Hruz T, Laule O, Szabo G, Wessendorp F, Bleuler S, et al (2008) Genevestigator v3: a reference expression database for the meta-analysis of transcriptomes. Adv Bioinformatics 2008: 420747. 19956698 10.1155/2008/420747 PMC2777001

[35] Zhang C, Guy CL (2006) In vitro evidence of Hsc70 functioning as a molecular chaperone during cold stress. Plant Physiol Biochem 44: 844–850. 17079155 10.1016/j.plaphy.2006.09.012

[36] Fuhrmann E, Bultema JB, Kahmann U, Rupprecht E, Boekema EJ, et al (2009) The vesicle-inducing protein 1 from *Synechocystis* sp. PCC 6803 organizes into diverse higher-ordered ring structures. Mol Biol Cell 20: 4620–4628. 19776353 10.1091/mbc.E09-04-0319 PMC2770949

[37] Lee BH, Henderson DA, Zhu JK (2005) The Arabidopsis cold-responsive transcriptome and its regulation by ICE1. Plant Cell 17: 3155–3175. 16214899 10.1105/tpc.105.035568 PMC1276035

[38] Brocchieri L, Conway de Macario E, Macario AJ (2008) hsp70 genes in the human genome: Conservation and differentiation patterns predict a wide array of overlapping and specialized functions. BMC Evol Biol 8: 19. 18215318 10.1186/1471-2148-8-19 PMC2266713

[39] Genevaux P, Georgopoulos C, Kelley WL (2007) The Hsp70 chaperone machines of *Escherichia coli*: a paradigm for the repartition of chaperone functions. Mol Microbiol 66: 840–857. 17919282 10.1111/j.1365-2958.2007.05961.x

[40] Schroda M (2004) The *Chlamydomonas* genome reveals its secrets: chaperone genes and the potential roles of their gene products in the chloroplast. Photosynth Res 82: 221–240. 16143837 10.1007/s11120-004-2216-y

[41] Sung DY, Vierling E, Guy CL (2001) Comprehensive expression profile analysis of the Arabidopsis Hsp70 gene family. Plant Physiol 126: 789–800. 11402207 10.1104/pp.126.2.789 PMC111169

[42] Ueguchi C, Kakeda M, Yamada H, Mizuno T (1994) An analogue of the DnaJ molecular chaperone in *Escherichia coli* . Proc Natl Acad Sci USA 91: 1054–1058. 8302830 10.1073/pnas.91.3.1054 PMC521452

[43] Perry SE, Li H-m, Keegstra K (1991) *In vitro* reconstitution of protein transport into chloroplasts. Methods Cell Biol 34: 327–344. 1943807 10.1016/s0091-679x(08)61688-x

[44] Jackson DT, Froehlich JE, Keegstra K (1998) The hydrophilic domain of Tic110, an inner envelope membrane component of the chloroplastic protein translocation apparatus, faces the stromal compartment. J Biol Chem 273: 16583–16588. 9632730 10.1074/jbc.273.26.16583

[45] Alonso JM, Stepanova AN, Leisse TJ, Kim CJ, Chen H, et al (2003) Genome-wide insertional mutagenesis of *Arabidopsis thaliana* . Science 301: 653–657. 12893945 10.1126/science.1086391

[46] Sakurai T, Satou M, Akiyama K, Iida K, Seki M, et al (2005) RARGE: a large-scale database of RIKEN Arabidopsis resources ranging from transcriptome to phenome. Nucleic Acids Res 33: D647–650. 15608280 10.1093/nar/gki014 PMC539968

[47] Seki M, Carninci P, Nishiyama Y, Hayashizaki Y, Shinozaki K (1998) High-efficiency cloning of Arabidopsis full-length cDNA by biotinylated CAP trapper. Plant J 15: 707–720. 9778851 10.1046/j.1365-313x.1998.00237.x

[48] Seki M, Narusaka M, Kamiya A, Ishida J, Satou M, et al (2002) Functional annotation of a full-length Arabidopsis cDNA collection. Science 296: 141–145. 11910074 10.1126/science.1071006

[49] Thompson JD, Gibson TJ, Plewniak F, Jeanmougin F, Higgins DG (1997) The CLUSTAL_X windows interface: flexible strategies for multiple sequence alignment aided by quality analysis tools. Nucleic Acids Res 25: 4876–4882. 9396791 10.1093/nar/25.24.4876 PMC147148

