# Supplementary material for: Evolution of Chloroplast J Proteins
Source: PLoS One. 2013 Jul 23;8(7):e70384. doi: 10.1371/journal.pone.0070384 (PMC3720927; doi:10.1371/journal.pone.0070384)
Supplement: Figure S1 — Phylogenetic relationship of chloroplast J proteins from Arabidopsis, rice, soybean and grape. A neighbor-joining tree was constructed using multiple sequence alignments of full-length polypeptide sequences of genes shown in Table 2. Bootstrap analysis was computed with 1,000 replicates and the values are shown on the branches. The eleven clades classified from analyses of Arabidopsis chloroplast J proteins are marked with different background colors. Three subclades of the Fd domain-containing J-protein clade are indicated. (PDF) [file pone.0070384.s001.pdf]

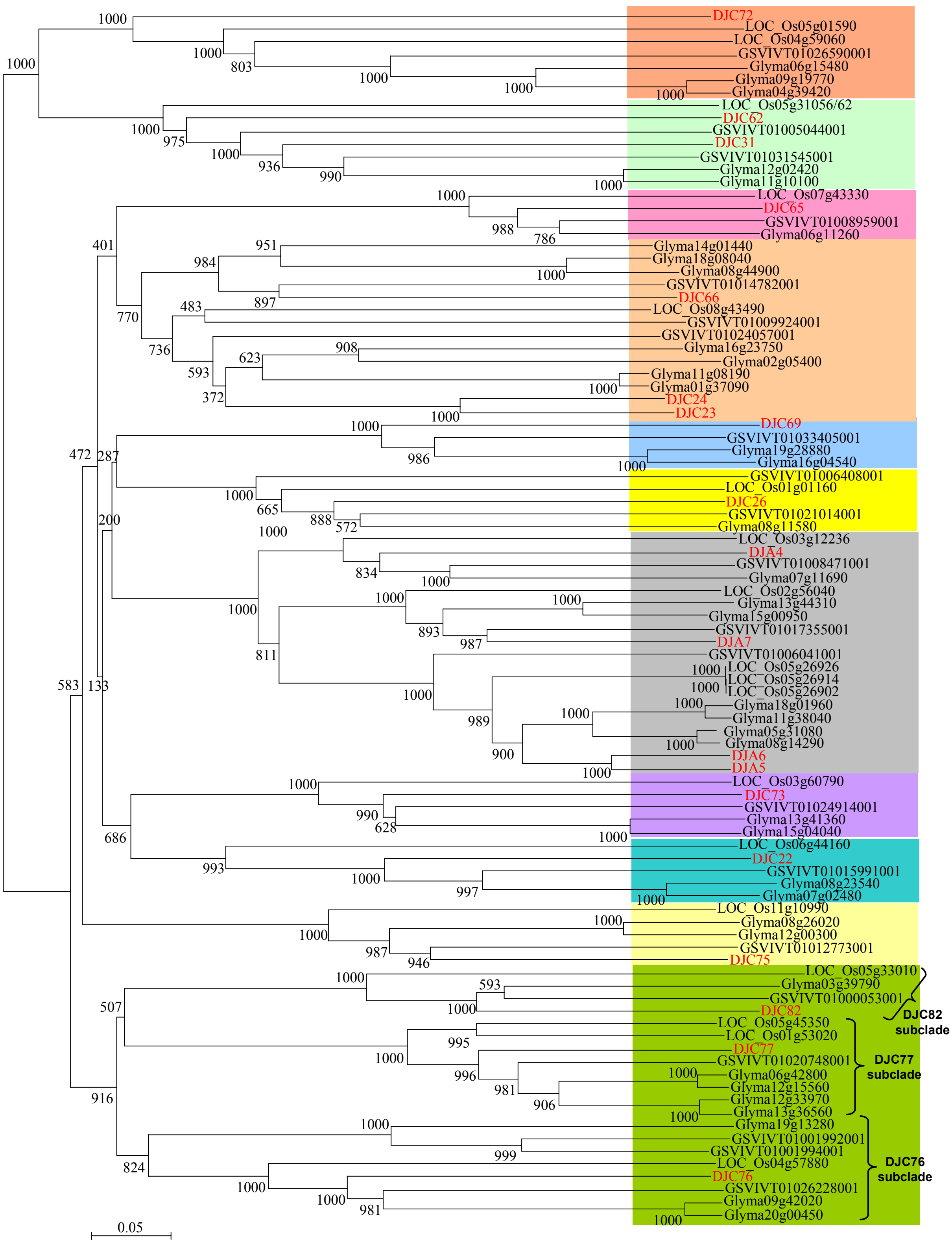

Figure S1. Phylogenetic relationship of chloroplast J proteins from Arabidopsis, rice, soybean and grape. A neighbor-joining tree was constructed using multiple sequence alignments of full-length polypeptide sequences of genes shown in Table 2. Bootstrap analysis was computed with 1,000 replicates and the values are shown on the branches. The eleven clades classified from analyses of Arabidopsis chloroplast J proteins are marked with different background colors. Three subclades of the Fd domain-containing J protein clade are indicated.
