# Supplementary material for: Evolution of Chloroplast J Proteins
Source: PLoS One. 2013 Jul 23;8(7):e70384. doi: 10.1371/journal.pone.0070384 (PMC3720927; doi:10.1371/journal.pone.0070384)
Supplement: Figure S2 — Sequence alignment of DJC31 homologs from Arabidopsis (DJC31), Brachypodium distachyon (Bradi2g27160), millet ( Setaria italica , SiPROV000210m), maize (GRMZM2G348697), sorghum (Sb09g018680), and the re-annotated rice DJC31, OsDJC31. OsDJC31 (GenBank accession: BK008486) was re-annotated from the continuous genomic region covered by loci LOC_Os05g31056 and LOC_Os05g31062. The J domain is underlined in blue. The position of the HPD tripeptide is indicated. (PDF) [file pone.0070384.s002.pdf]

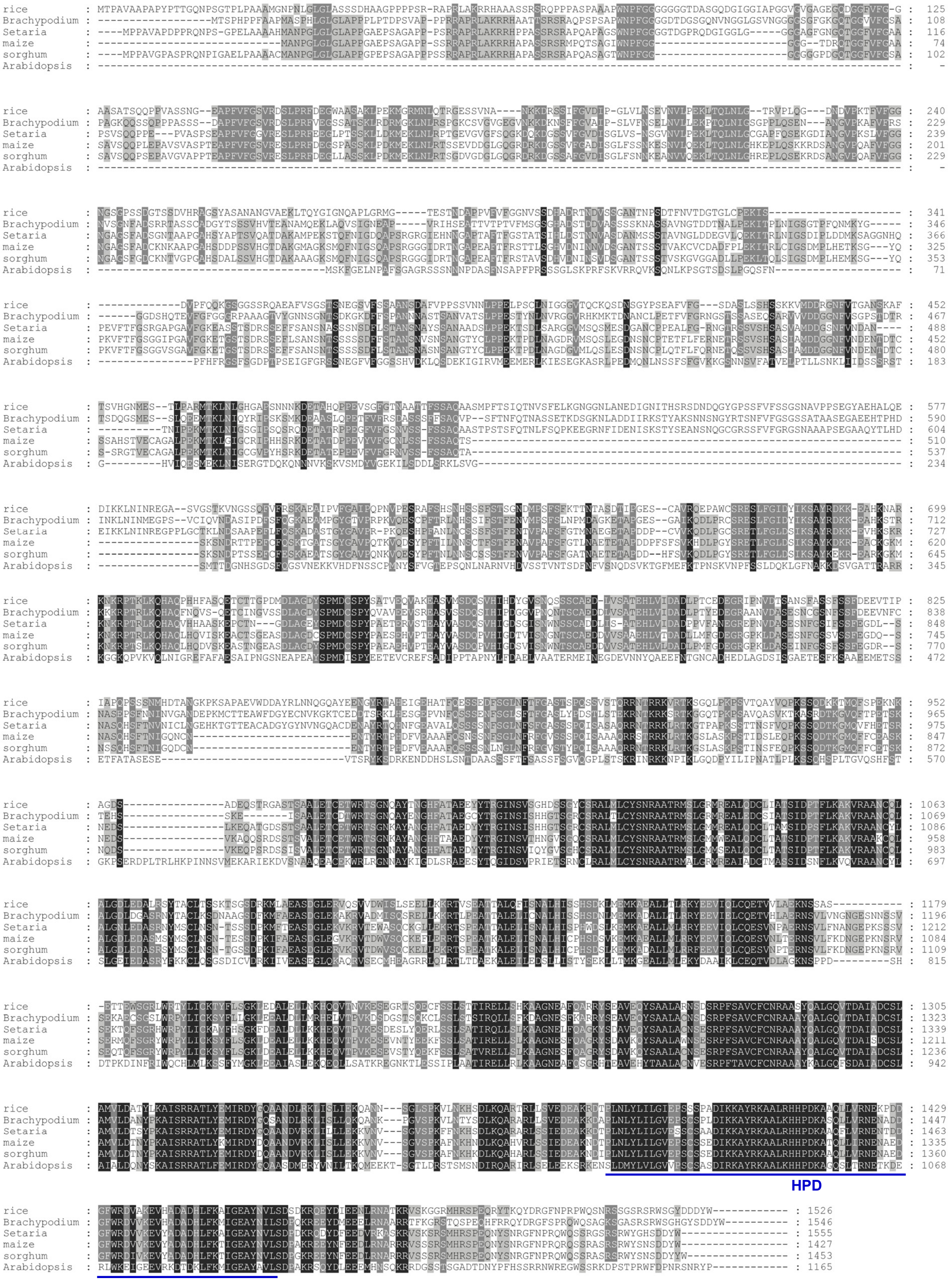

Figure S2. Sequence alignment of DJC31 homologs from Arabidopsis (DJC31), *Brachypodium distachyon* (Bradi2g27160), millet (*Setaria italica*, SiPROV000210m), maize (GRMZM2G348697), sorghum (Sb09g018680), and the re-annotated rice DJC31 homolog, OsDJC31.

OsDJC31 (GenBank accession: BK008486) was re-annotated from the continuous genomic region covered by loci LOC_Os05g31056 and LOC_Os05g31062. The J domain is underlined in blue. The position of the HPD tripeptide is indicated.
