# Supplementary material for: Evolution of Chloroplast J Proteins
Source: PLoS One. 2013 Jul 23;8(7):e70384. doi: 10.1371/journal.pone.0070384 (PMC3720927; doi:10.1371/journal.pone.0070384)
Supplement: Figure S3 — Sequence alignment of re-annotated rice DJC82 homolog with DJC82 homologs from Arabidopsis (DJC82), soybean (Glyma03g39790), grape (GSVIVT01000053001), Selaginella (g73652), and Physcomitrella (Ppls137_288V6.1). The rice DJC82 homologue, OsDJC82 (GenBank accession: BK008487), was re-annotated from original annotation for locus LOC_Os05g33010. The J domain is underlined in blue. The position of the HPD tripeptide is indicated. (PDF) [file pone.0070384.s003.pdf]

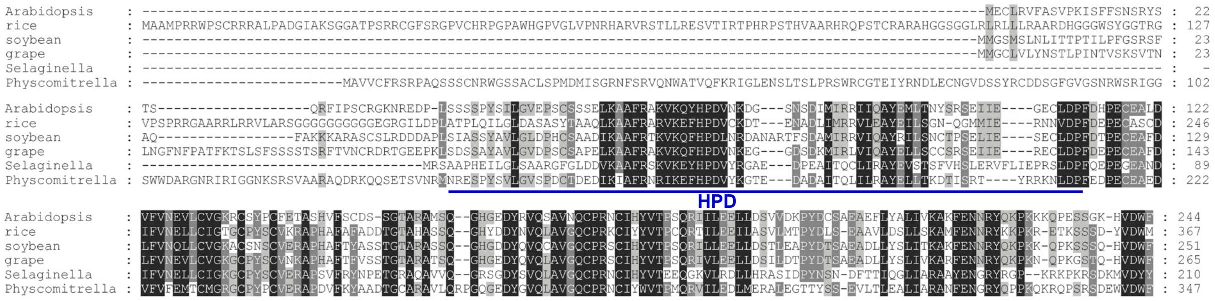

Figure S3. Sequence alignment of re-annotated rice DJC82 homolog OsDJC82 with DJC82 homologs from Arabidopsis (DJC82), soybean (Glyma03g39790), grape (GSVIVT01000053001), *Selaginella* (g73652), and *Physcomitrella* (Ppls137_288V6.1). OsDJC82 (GenBank accession: BK008487) was re-annotated from original annotation for locus LOC_Os05g33010. The J domain is underlined in blue. The position of the HPD tripeptide is indicated.
