# Supplementary material for: Evolution of Chloroplast J Proteins
Source: PLoS One. 2013 Jul 23;8(7):e70384. doi: 10.1371/journal.pone.0070384 (PMC3720927; doi:10.1371/journal.pone.0070384)
Supplement: Figure S4 — Sequence alignment of re-annotated Selaginella DJC73 homolog with DJC73 homologs from Arabidopsis (DJC73), rice (LOC_Os03g60790), soybean (Glyma13g41360), grape (GSVIVT01024914001), and Physcomitrella (Ppls137_288V6.1). The Selaginella DJC73 homolog, SmDJC73 (GenBank accession: BK008488), was re-annotated from original annotation for locus g8874. The J domain is underlined in blue. The position of the HPD tripeptide is indicated. (PDF) [file pone.0070384.s004.pdf]

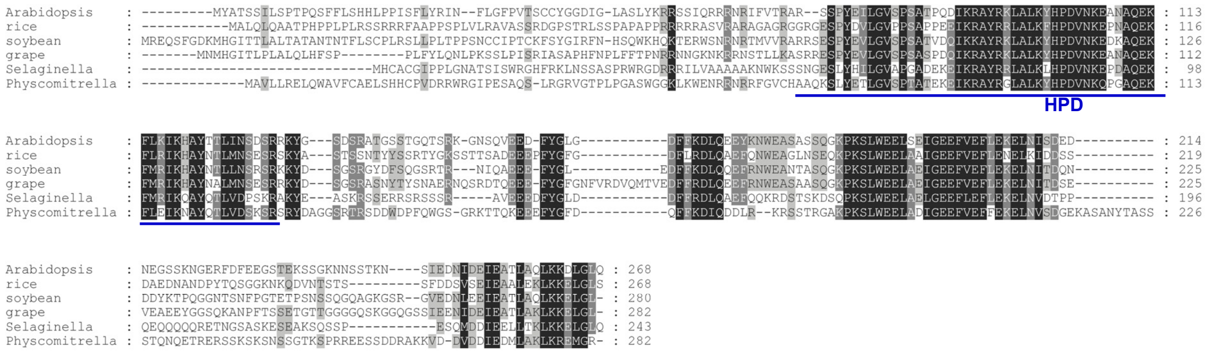

Figure S4. Sequence alignment of re-annotated *Selaginella* DJC73 homolog with DJC73 homologs from Arabidopsis (DJC73), rice (LOC_Os03g60790), soybean (Glyma13g41360), grape (GSVIVT01024914001), and *Physcomitrella* (Ppls137_288V6.1). The *Selaginella* DJC73 homolog, SmDJC73 (GenBank accession: BK008488) was re-annotated from original annotation for locus g8874. The J domain is underlined in blue. The position of the HPD tripeptide is indicated.
