# Supplementary material for: Evolution of Chloroplast J Proteins
Source: PLoS One. 2013 Jul 23;8(7):e70384. doi: 10.1371/journal.pone.0070384 (PMC3720927; doi:10.1371/journal.pone.0070384)
Supplement: Table S1 — Homologs of Arabidopsis chloroplast J protein in seven green algal genomes. (PDF) [file pone.0070384.s005.pdf]

Table S1. Homologs of Arabidopsis chloroplast J protein in seven green algal genomes.

Arabidopsis thaliana	*Volvox carteri*	*Chlamydomonas reinhardtii*	*Micromonas pusilla* RCC299	*Micromonas pusilla* CCMP1545	*Ostreococcus tauri*	*Ostreococcus lucimarinus*	*Ostreococcus sp.* RCC809
DJA4<br>DJA5<br>DJA6<br>DJA7	Vocar20001038m.g<br>Vocar20002133m.g	CDJ1<br>CDJ6	MICPUN_108923	MICPUCDRAFT_23733	Ot10g00240	OSTLU_12940<br>OSTLU_89287	38086<br>42776
DJC22	-	-	-	-	-	-	-
DJC23<br>DJC24<br>DJC66	-	-	-	-	-	-	-
DJC26	-	-	-	-	-	-	-
DJC31<br>DJC62	Vocar20001893m.g	Cre02.g108800	MICPUN_55539<br>MICPUN_58681<br>MICPUN_64027	MICPUCDRAFT_69675	Ot01g03500<br>Ot09g00550<br>Ot05g03750	OSTLU_8481<br>OSTLU_31834	55618
DJC65	-	-	-	-	-	-	-
DJC69	-	-	-	-	-	-	-
DJC72	-	-	-	-	-	-	-
DJC73	Vocar20012653m.g	CDJ2	between loci MICPUN_92934 and MICPUN_55099[a]	MICPUCDRAFT_49920	Ot03g04930	OSTLU_9681	57837
DJC75	-	-	-	-	-	-	-
DJC76<br>DJC77<br>DJC82	Vocar20003080m.g<br>Vocar20002706m.g	CDJ3<br>CDJ4<br>CDJ5	MICPUN_64158<br>MICPUN_57928	MICPUCDRAFT_70264<br>MICPUCDRAFT_58089	Ot01g01190	OSTLU_9697<br>OSTLU_7956	15295<br>27952

[a]*Micromonas* DJC73 homolog was found to locate in the intergenic region between loci MICPUN_92934 and MICPUN_55099
