# Supplementary material for: Evolution of Chloroplast J Proteins
Source: PLoS One. 2013 Jul 23;8(7):e70384. doi: 10.1371/journal.pone.0070384 (PMC3720927; doi:10.1371/journal.pone.0070384)
Supplement: Table S2 — J proteins in Synechocystis sp. PCC 6803. (PDF) [file pone.0070384.s006.pdf]

Table S2. J proteins in *Synechocystis* sp. PCC 6803.

Type	J protein
I	Sll0897
II	Sll1933<br>Slr0093
III	Sll0909<br>Sll1011<br>Sll1384<br>Sll1666
