# Supplementary material for: Evolution of Chloroplast J Proteins
Source: PLoS One. 2013 Jul 23;8(7):e70384. doi: 10.1371/journal.pone.0070384 (PMC3720927; doi:10.1371/journal.pone.0070384)
Supplement: Table S3 — Information of J-protein clones used in this study. (PDF) [file pone.0070384.s007.pdf]

Table S3. Information of J-protein clones used in this study.

J-protein clones	clone requested	primer name		RNA polymerase used for in vitro translation
		forward primer	reverse primer	
DJA4	-	DJA4-S	DJA4-AS	T7
DJA5	U13727	-	-	T3
DJA6	-	DJA6-HindIII-5'	DJA6-EcoRI-3'	T7
DJA7	-	DJA7-S	DJA7-AS	T7
DJC23	U83438	-	-	T3
DJC24	U12528	-	-	T3
DJC26	U09577	-	-	T3
DJC31	-	KpnI-DJC31-F1	ClaI-DJC31-R1	SP6
DJC31-G184x	-	KpnI-DJC31-F1	DJC31-G184x-R-ClaI	SP6
DJC42	-	PstI-DJC42-F1	KpnI-DJC42-R1	SP6
DJC62	RAFL_08-18-019	-	-	T7
DJC62-R180x	-	KpnI-DJC62-F1	DJC62-R180x-R1-ClaI	SP6
DJC65	-	SphI-DJC65-F1	XbaI-DJC65-R1	SP6
DJC66	-	XhoI-DJC66-F1	XbaI-DJC66-R1	SP6
DJC69	-	XhoI-DJC69-F1	XbaI-DJC69-R1	SP6
DJC72	-	KpnI-DJC72-F1	BglII-DJC72-R1	SP6
DJC72MM		DJC72-diMet-F1	DJC72-diMet-R1	SP6
DJC73	U86815	-	-	T3
DJC73MM	-	DJC73-KpnI-F1	DJC73-diMet-ClaI-R1	SP6
DJC75	BX829231	-	-	SP6
DJC76	U87767	-	-	T3
DJC77	U21285	-	-	T3
DJC78	U11662	-	-	SP6
DJC78MM		DJC78-diMet-F1	DJC78-diMet-R1	SP6
DJC82	-	HindIII-DJC82-F1	BglII-DJC82-R1	SP6

Accession numbers are listed for clones requested from various centers. Names of primers (see Table S4 for primer sequences) used are provided for clones isolated in this study.
