# Supplementary material for: Evolution of Chloroplast J Proteins
Source: PLoS One. 2013 Jul 23;8(7):e70384. doi: 10.1371/journal.pone.0070384 (PMC3720927; doi:10.1371/journal.pone.0070384)
Supplement: Table S4 — Sequences of primers used in this study. (PDF) [file pone.0070384.s008.pdf]

Table S4. Sequences of primers used in this study.

clone name	Primer Sequence (from 5' to 3')
DJA4-S	gaatgagatataacaatggctgcaatgg
DJA4-AS	ccatgaggccttaagtttctttc
DJA6-HindIII-5'	ctcgaagcttttccgtcgtccacca
DJA6-EcoRI-3'	ttcggaattctaatcatctactggtgc
DJA7-S	gattcctagtcgtcaacaatggctgccctc
DJA7-AS	gcaagacctgttagttgaagatgg
KpnI-DJC31-F1	cccggtaccatgagcaagttcggcgaattgaatc
ClaI-DJC31-R1	cccatcgatttacgggtatctgtttgatcggtttg
DJC31-G184x-R-ClaI	cccatcgattcatgtactcctcgaagatgaatca
PstI-DJC42-F1	ccccagctgatggactggaataaagaagaggcat
KpnI-DJC42-R1	cccggaaccctatgggttctttaagttgagaccca
Kpn-DJC62-F1	cccggtaccatgtctcctgcggcggtggagatt
DJC62-R180x-R1-ClaI	cccatcgattcagatgttttcccgatcattatca
SphI-DJC65-F1	cccgcatgcatgagcaatcttcgagctatctgtaga
XbaI-DJC65-R1	ccctctagattactttgctgcttcttcctcttctgct
XhoI-DJC66-F1	cccctcgagatggctggaaccctagttaactccg
XbaI-DJC66-R1	ccctctagatcaccaacactgatccgtctcccat
XhoI-DJC69-F1	cccctcgagatgcagactcaccttttggtggg
XbaI-DJC69-R1	ccctctagatcaggttgagtcgacttgaagtttga
KpnI-DJC72-F1	cccggtaccatgtttagattgagaagtctcttgt
BglII-DJC72-R1	cccagatcttcaagcgagtgcagaacagagggac
DJC72-diMet-F1	ccctctgttctgcactcgctatgatgtgaagatctgccggtctccc
DJC72-diMet-R1	gggagaccggcagatcttcacatcatagcgagtgcagaacagaggg
DJC73-KpnI-F1	cccggtaccatgtatgcaacatcttcgattctgt
DJC73-diMet-ClaI-R1	cccatcgatttacatcatttgcaagccaagatctttcttcaat
DJC78-diMet-F1	aacaggaaataaaagagaagatgatgtgattttttttggtaagcga
DJC78-diMet-R1	tcgcttaccaaaaaaaatcacatcatcttctcttttatttcctgtt
HindIII-DJC82-F1	cccaagcttatggaatgtcttcgtgtatttgcttc
BglII-DJC82-R1	cccagatcttcagaaccagtctacatgttttcct
